# Introducing care navigator based, non‐pharmacologic, supplemental care model for the treatment of behavioral and psychological symptoms of dementia in Addis Ababa, Ethiopia

**DOI:** 10.1002/alz70858_100239

**Published:** 2025-12-25

**Authors:** Selam Aberra Yoseph, Mekdes Demissie

**Affiliations:** ^1^ Addis Ababa University, Addis Ababa, Ethiopia; ^2^ Addis Ababa University, Addis Ababa, CA, Ethiopia; ^3^ Global brain health institute, SAN FRANCISCO, CA, USA

## Abstract

**Background:**

Behavioral and psychological symptoms of dementia (BPSDs) affect most individuals with dementia in the course of their illness. Non‐pharmacological interventions, particularly those targeting family caregivers, are recommended as first‐line treatments. Care navigation is a promising approach, where trained navigators collaborate with patients and caregivers to provide education on behavioral management, coping mechanisms, and communication skills, leading to better outcomes.

**Method:**

Four nurses were trained as care navigators through theoretical and practical sessions. Fifty patient‐caregiver dyads from the Neurology and Psychiatry outpatient clinics at Tikur Anbessa Specialized Hospital were recruited. We included any cause for the dementia and all severity levels.

The navigators introduced a non‐pharmacologic supplemental care to the dyads, on top of their usual care, by following a manual that was prepared by adapting a toolkit used for a similar study in the United States called the Care Ecosystem.

The dyads came monthly for 6 months receiving education and training on different topics including what dementia is, how to communicate with people living with dementia, safety protocols, advance care planning and caregiver wellbeing. Support group sessions facilitated by the clinical team allowed caregivers to share experiences and learn from each other.

**Result:**

The mean age of patients was 65.1 years, with a majority being female (66%) and married (70%). The primary caregivers were mostly children (40%) and spouses (38%). At baseline, most patients (84%) had a Neuropsychiatric Inventory (NPI) score >3, indicating prevalent symptoms like agitation, depression, irritability, and motor behavior. Additionally, 78% of participants were on psychotropic medication, with 20% using multiple medications. Caregivers reported high levels of perceived stigma (86%) and burden (mean score of 5.1), and nearly half showed depressive symptoms (mean PHQ‐9 score of 7.8).

**Conclusion:**

In conclusion, care navigator based model is a feasible approach for managing dementia in low‐ and middle‐income countries like Ethiopia, offering a promising alternative to pharmacological treatments.

